# Quantifying prenatal and postnatal mortality in extreme early onset fetal growth restriction: A systematic review and incidence analysis

**DOI:** 10.1002/pmf2.70034

**Published:** 2025-05-24

**Authors:** Shohra Qaderi, Giulia Bonanni, Mohammadamin Parsaei, Ehsan Rojhani, Satjeet Deol Chauhan, Eyal Krispin, Dario O. Fauza, Anna L. David, Kjersti Aagaard, Alireza A. Shamshirsaz

**Affiliations:** ^1^ Fetal Care and Surgery canter, Division of Fetal Medicine and Surgery Boston Children's Hospital, Harvard Medical School Boston Massachusetts USA; ^2^ Department of Women, Children, and Public Health Sciences IRCCS Agostino Gemelli University Polyclinic Foundation, Catholic University of the Sacred Heart Rome Italy; ^3^ Breastfeeding Research Center, Family Health Research Institute Tehran University of Medical Sciences Tehran Iran; ^4^ Department of Obstetrics and Gynecology BronxCare Health System/Icahn School of Medicine at Mount Sinai Bronx New York USA; ^5^ Department of Surgery Boston Children's Hospital/Harvard Medical School Boston Massachusetts USA; ^6^ UCL Elizabeth Garrett Anderson Institute for Women's Health University College London London UK; ^7^ HCA Healthcare and HCA Research Institute Nashville Tennessee USA; ^8^ National Director of Perinatal Research and HCA Healthcare Gulf Coast Division & HCA Texas Maternal Fetal Medicine Houston Texas USA

**Keywords:** fetal growth restriction, intrauterine growth restriction, IUGR, maternal health, perinatal outcomes

## Abstract

**Background and Objective:**

Extreme early‐onset fetal growth restriction (EE‐FGR) is associated with significant perinatal morbidity and mortality. As short‐ and long‐term prognoses are influenced by both gestational age and severity of FGR, quantifying risk is inherently challenging. We hypothesized that a meta‐analysis would provide quantifiable estimates of perinatal outcomes in pregnancies complicated by EE‐FGR.

**Methods:**

We searched PubMed, Scopus, Embase, and Web of Science databases for relevant studies published from inception to March 2024. We included all original studies involving singleton pregnancies with estimated fetal weights (EFWs) or abdominal circumferences < third centile or EFWs < 600 g, between 20 + 0 and 26 + 6 weeks of gestation. Studies were limited to fetuses with no known chromosomal, genetic, or major structural abnormalities. The National Institute of Health Quality Assessment Tool was utilized for the risk of bias assessment. Incidence meta‐analyses were performed with proportions (percentages) calculated for each outcome using a random‐effects model.

**Results:**

Five cohort studies (three prospective and two retrospective), comprising a total of 614 pregnancies, were included in the review. Two studies had overlapping populations; in such cases, the study containing the most relevant variables was selected for analysis. Four studies demonstrated good quality, and one was of fair quality. Pregnancy loss occurred at a rate of 39% (95% confidence interval [CI]: 29%–49%). Among live births, neonatal death was observed in 14% (95% CI: 4%–24%). Preterm birth (<37 weeks) was reported in 68% of cases (95% CI: 40%–96%), with very preterm birth (<32 weeks) accounting for 61% (95% CI: 34%–88%) of these, and extreme preterm birth (<28 weeks) comprising 25%. Additionally, 29% (95% CI: 20%–39%) of pregnancies were complicated by preeclampsia and/or hemolysis, elevated liver enzyme levels, and low platelet levels (HELLP) syndrome, with high heterogeneity (*I* ^2^ > 50%) across outcomes.

**Conclusion:**

Our meta‐analyses quantitated the incidence of adverse fetal, neonatal, and maternal outcomes with EE‐FGR, with the risk of pregnancy loss or neonatal death approaching nearly 50%, and among survivors, more than two thirds were born preterm. These findings provide valuable data for counseling families facing EE‐FGR, though high heterogeneity highlights the need for further research.

**Trial Registration:**

PROSPERO database [CRD42019120930].

## 1 INTRODUCTION

Fetal growth restriction (FGR), previously known as intrauterine growth restriction (IUGR), is a leading cause of perinatal morbidity and mortality. It affects approximately 8% of pregnancies and is associated with 50% of stillbirths [1]. The severity and timing of FGR onset are critical, with early‐onset FGR (before 32 weeks of gestation) posing unique challenges. Management of early‐onset FGR often requires balancing the risks associated with the restrictive growth itself against those of early delivery, which introduces additional complications related to extreme prematurity, impacting both short‐ and long‐term outcomes [2, 3].

It has been well established that FGR is one of the most frequent and common independent risk factors for poor outcomes in infants [4]. When severe and early‐onset FGR and subsequent often extreme preterm birth (PTB) impose a broad spectrum of adverse pregnancy outcomes, including fetal and neonatal death [5, 6], necrotizing enterocolitis [5], respiratory complications, neurodevelopmental disorders [7, 8, 9, 10], and lifelong health risks such as obesity and hypertensive diseases [11, 12, 13]. The most challenging early FGR occurs when the fetus is very small at the mid‐gestation scan with or without fetal Doppler abnormalities. A previous multicenter study of severe early‐onset FGR has defined this as an estimated fetal weight <third percentile and <600 g, between 20 + 0 and 26 + 6 weeks of gestation [14]. These are also the patients who may be most suitable for clinical trials of interventions to improve fetal growth and/or perinatal outcome.

Quantifying perinatal mortality is challenging due to the complex interplay between gestational age and the extent of FGR. However, understanding the rates of adverse outcomes is crucial for patient counseling and care management. In this context, we hypothesized that a meta‐analysis would provide quantifiable estimates of perinatal outcomes related to extreme early‐onset FGR (EE‐FGR).

## 2 METHODS

### 2.1 Protocol registration

We adhered to the Preferred Reporting Items for Systematic Reviews and Meta‐Analyses (PRISMA) statement guidelines 2020 for conducting this systematic review and meta‐analysis [15]. The protocol for this study was registered on PROSPERO (CRD42024570738).

### 2.2 Database sources and search strategy

To identify relevant studies for inclusion in this study, we conducted a literature search for articles published up to March 2024 across multiple electronic databases (PubMed, Embase, and Web of Science) using relevant keywords. The search strategy included combinations of terms such as “Fetal Growth Restriction,” “IUGR,” “Intrauterine Growth Restriction,” and “Fetal Growth Retardation.” To ensure comprehensive coverage, we did not impose any restrictions on publication dates, and we also searched conference abstracts and the reference lists of included studies.

### 2.3 Inclusion and exclusion criteria

We included studies that reported outcomes associated with EE‐FGR. The inclusion criteria were as follows:

- Studies involving live singleton fetuses with an estimated fetal weight (EFW) < 600 g and <third percentile for gestational age or abdominal circumference (AC) < third percentile.
- Diagnoses made between 20 + 0 and 26 + 6 weeks, based on ultrasound and/or last menstrual period.
- Sufficient reporting of outcomes, including at least pregnancy loss.

Studies that met any of the following conditions were excluded:

- Reviews, comments, and case reports.
- Studies conducted on nonhuman subjects, such as animal models.
- Ongoing studies.
- Conference abstracts.
- Twin pregnancies, known abnormal karyotype at enrolment, known major fetal structural abnormalities, indication for immediate delivery, any medical or psychiatric condition compromising participation, maternal HIV or hepatitis B or C infection, premature preterm rupture of membranes before enrolment
- Studies that do not provide relevant outcome data on pre‐ and postnatal mortality and morbidity.

### 2.4 Study selection and data extraction process

#### 2.4.1 Selection process

After retrieving citations from electronic databases, we removed duplicates using EndNote [16]. This step included reviewing data from the reported studies to look for duplication. The records were then uploaded and screened in two steps using Rayyan software [17]. First, two authors (G.B. and M.P.) independently screened the titles and abstracts of all references to assess their relevance to our meta‐analysis. Next, the full text of relevant articles was evaluated for final eligibility. Any disagreements between the two reviewers were resolved by a third reviewer (S.Q. or E.R.).

#### 2.4.2 Data extraction

Detailed information was systematically extracted from all eligible studies, including the first author's name, year of publication, sample size, basic characteristics, study population, study design, study duration, registration number, and outcomes. Two independent authors (M.P. and E.R.) manually performed this process using an online data extraction form created in Google Sheets (Google LLC).

### 2.5 Assessed outcomes

The primary outcomes of this study were: (I) pregnancy loss (prenatal mortality), defined as any death during the antenatal period, including IUFD (intrauterine fetal demise), TOP (termination of pregnancy), and stillbirth, as these terms were defined in the original studies themselves. (II) Neonatal death, defined as death occurring within the first 30 days of life.

The secondary outcomes were: (I) preeclampsia and/or HELLP syndrome; (II) cesarean delivery; (III) PTB (delivery at <37 gestational weeks); (IV) extreme PTB (delivery at <28 gestational weeks); and (V) very or extreme PTB < 32 gestational weeks. We also included outcomes deemed important to patients and clinicians Spencer et al. [18], including fetal or neonatal death and fetal death or delivery < 28 gestational weeks.

### 2.6 Grading the quality of evidence

Study quality was assessed using the National Institute of Health (NIH) Quality Assessment Tool for Observational Cohort and Cross‐Sectional Studies [19]. The certainty of evidence (e.g., confidence in results) was classified as good, fair, or poor.

### 2.7 Data synthesis and publication assessment

The data collected from each included study were transferred from the online data extraction form to the STATA 18.0 software (StataCorp LP) [20]. Subsequently, prevalence meta‐analyses were conducted to examine the pooled prevalence/occurrence rate of each outcome reported by at least three studies. Given that two studies shared a dataset [18, 21], the larger patient count for each mutually reported variable was selected for the meta‐analysis.

The effect size for each analysis was presented as a proportion (and percentage) along with a 95% confidence interval (CI) around the summary estimate. Pooled prevalence estimates were generated using a random‐effects model with the restricted maximum likelihood method. Forest plots were generated to visually display the effect sizes from individual studies, the pooled effect size, and the degree of heterogeneity. Heterogeneity across each meta‐analysis was evaluated using the *I* ^2^ index, with *I* ^2^ > 50% indicating high heterogeneity and *I* ^2^ < 50% indicating low heterogeneity among the analyzed studies. Also, sensitivity analyses were conducted for analyses including more than three studies, employing a leave‐one‐out meta‐analysis to evaluate the impact of each individual study.

Furthermore, univariate meta‐regression analyses were performed using a random‐effects model to investigate the extent to which four specific variables—publication year (before and after 2020), study design (prospective vs. retrospective), study setting (single‐center vs. multicenter), and diagnostic criteria for EE‐FGR (EFW or AC)—could account for the observed heterogeneity in the analyses of the primary outcomes (pregnancy loss and neonatal death). In addition, for the neonatal death outcome, the gestational age at delivery was also included in our meta‐regression analysis. For variables that explained at least 30% of the observed heterogeneity (*R* ^2^ > 30%), separate subgroup analyses were conducted using the same random‐effects model.

To assess potential publication bias in the studies analyzed, two objective methods were employed following each meta‐analysis: the nonparametric rank correlation (Begg) test [22] and the regression‐based Egger test for small study effects [23]. A *p*‐value of less than 0.05 was considered indicative of potential publication bias. Additionally, funnel plots were generated to provide a visual assessment of the asymmetry in the calculated effect sizes across the included studies to subjectively evaluate the potential presence of publication bias. In cases where publication bias was detected, the trim‐and‐fill method by Duval and Tweedie was used to address asymmetries in effect sizes and impute missing studies on either the left (L0) or right (R0) side of the funnel plots [24]. New pooled prevalence's for the analyzed outcomes were then calculated after imputing the missing studies.

## 3 RESULTS

### 3.1 Search results and study selection

**FIGURE 1:**
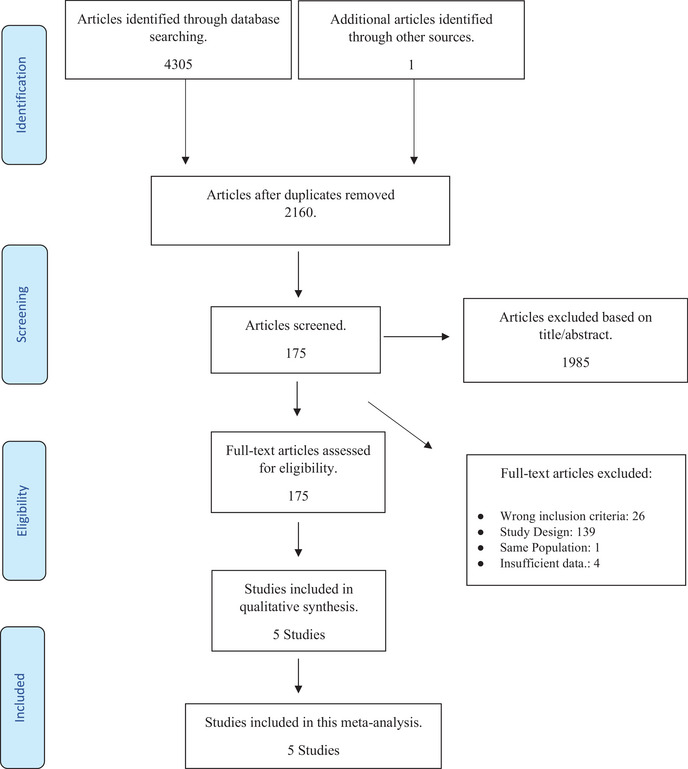
Flow diagram of the study selection process.

Our systematic literature search identified 4305 potentially relevant studies from the electronic databases. After removing duplicates, 2160 records remained. Screening of titles and abstracts resulted in 175 studies that met eligibility criteria. A full‐text assessment was then conducted, leading to the exclusion of 170 studies with specific reasons for exclusion (Figure 1). Therefore, five studies were included for qualitative and quantitative synthesis.

### 3.2 Characteristics of included studies

A total of five studies published between 2007 and 2023 were systematically reviewed. Among these, one study was conducted in the United States [25], one in the United Kingdom [5], one in France [26], and two were multicenter studies from the United Kingdom, Germany, Sweden, and Spain [18, 21]. Three of the included studies employed a prospective cohort design [18, 21, 25], while the remaining two had a retrospective cohort design [5, 26]. Sample sizes ranged from 13 to 245, culminating in a total of 614 cases of EE‐FGR examined. Of note, two of the included studies had significant population overlap, as both were part of the EVERREST study cohort. To avoid duplication and ensure consistency in data extraction, only the study that reported the most relevant clinical variables (Spencer and Lingam) was retained for analysis [14, 21].

The diagnosis of EE‐FGR was based on predefined criteria: EFW below the third percentile in two studies [25, 26], EFW below the third percentile and less than 600 g in two studies [18, 21], and AC below the third percentile in one study [5]. Table 1 provides detailed information on the sample characteristics and findings of each included study.

**TABLE 1:** Study characteristics and maternal and neonatal outcome.

Study	Institute	Country	Timeline	Sample size	GA assessment method	GA at diagnosis (mean)	GA at delivery (mean)	Nulliparous; *n* (%)	Chronic medical illness in pregnant person; *n* (%)	HELLP; *n* (%)	Oligohydramnios, *n* (%)	EDF absent or reversed *n* (%)	Cesarian delivery; *n* (%)	Birthweight mean (g)	Preterm birth; *n* (%)	Prenatal mortality; *n* (%)	Neonatal death; *n* (%)
Mari et al. [25]	Wayne State University	USA^b^	‐	13	LMP and/or scan	25.3	26.9	‐	10 (76.9)	4 (30.7)	‐	‐	‐	491	9 (100)	4 (30.7)	4 (44.4)
Lawin‐O'Brien et al. [5]	Queen Charlotte's and Chelsea Hospital (QCCH); Guy's and St Thomas’ Hospital (GSTH); St George's Hospital (SGH)	UK^c^	2000–2015	245	First trimester ultrasound (11–13+6 GA)	23.59	27.95	132 (54)	‐	81 (33)	91 (39)	77 (57.45)	98 (79.67)	1064.88	86 (69.9)	122 (49.79)	22 (17.8)
Dap et al. [26]	Nancy Maternity University Hospital	France^c^	2013–2020	98	First trimester ultrasound (11–13+6 GA)	22.7	31.5	60 (61.2)	16 (16.2)	18 (18.4)	23 (23.34)	13 (13)	23 (38.3)		17 (27.4)	36 (36.7)	2 (3.22)
Spencer et al. [18]^a^	University College London Hospital	UK, Germany, Sweden, Spain^b^	2014–2020	123	LMP (confirmed by scan at 16 GA)	23.71	28.3	80 (65)	16 (13)	41 (36)	‐	43 (35)	79 (89)		‐	‐	‐
Lingam et al. [21]^a^	University College Hospital London; University Medical Center Hamburg‐Eppendorf; Maternal‐Fetal Unit Hospital Clinic de Barcelona; Lund University Hospital	UK, Germany, Spain, Sweden^b^	2014–2020	135	LMP (confirmed by scan at 16 GA)		31.4	‐	‐	‐	‐	‐	‐	1149	74 (80)	135 (42)	12 (13)

Abbreviations: EDF, end‐diastolic flow; GA, gestational age; HELLP, hemolysis, elevated liver enzymes, and low platelet levels; LMP, last menstrual period; *n*, number.

^a^ The populations in the two studies significantly overlapped as part of the EVERREST study, and the analysis focused on the study that presented the relevant variables.

^b^ Prospective cohort study.

^c^ Retrospective cohort study.

### 3.3 Risk of bias and quality assessment

The results of the quality assessment are detailed in Table S1. None of the included studies had a poor overall quality rating. Four studies were rated as good quality [5, 18, 21, 26], and one was rated as having fair overall quality [25].

### 3.4 Meta‐analysis

#### 3.4.1 Primary outcomes

*Pregnancy loss*: By pooling data from four studies [5, 21, 25, 26], encompassing a total of 491 maternal‐fetal pairs with EE‐FGR, we estimated an overall pregnancy loss rate of 39% (95% CI: 29%–49%, *I* ^2^ = 76.28%), with leave‐one‐out analysis yielding rates from 33% to 42% (Figure S1). No potential publication bias was found, as evidenced by Egger's and Begg's *p*‐values of 0.454 and 1.000. Meta‐regression analyses indicated that publication year, study design, and EE‐FGR diagnostic criteria significantly explained the observed heterogeneity (*R* ^2^ = 98.16%, 40.92%, and 100.0%, respectively; Table S2). Subgroup analyses revealed pooled pregnancy loss rates of 44% (95% CI: 27%–61%) for studies published before 2020 and 33% (95% CI: 27%–39%) for those after 2020 (Figure S2). Rates were 31% (95% CI: 24%–39%) for retrospective studies and 44% (95% CI: 31%–57%) for prospective studies (Figure S3). Additionally, rates were 33% (95% CI: 27%–39%) for studies defining EE‐FGR by EFW and 50% (95% CI: 44%–56%) for those using AC (Figure S4).

*Neonatal death*: By pooling data from four studies [5, 21, 25, 26], encompassing a total of 287 live births, neonatal death occurred in 14% of cases (95% CI: 4%–24%, *I* ^2^ = 84.79%), with leave‐one‐out analysis yielding rates from 11% to 17% (Figure S5). Furthermore, Egger's test indicated potential publication bias (*p*‐value = 0.025), prompting the use of the trim‐and‐fill method. After imputing one missing study on the left side of the funnel plot, the recalculated pooled neonatal death rate was 9% (95% CI: 6%–12%). In further meta‐regression analyses, only gestational age at delivery significantly explained the observed heterogeneity (*R* ^2^ = 46.32%). Based on this finding, a subgroup analysis was conducted (Table S3). The analysis revealed a pooled neonatal death rate of 26% (95% CI: 2%–50%) for cohorts with a mean gestational age below 30 weeks and 8% (95% CI: 0%–17%) for those with a mean gestational age above 30 weeks (Figure S6).

#### 3.4.2 Secondary outcomes

The pooled PTB rate was calculated as 68% of cases (95% CI: 40%–96%, *I* ^2^ = 96.94%; Figure S7). Also, a pooled incidence of 61% (95% CI: 34%–88%, *I* ^2^ = 96.36%) was calculated for the very preterm PTB (Figure S8). Figure S9 provides a detailed overview of preterm deliveries based on definitions from various studies, categorized by different gestational ages. This figure presents the distribution of gestational ages at delivery for fetuses that survived until birth. Although the gestational age at delivery varies significantly across studies due to different cut‐offs, this figure offers an overall understanding of PTB.

Maternal adverse outcomes were also prevalent, with preeclampsia/HELLP syndrome affecting 29% of pregnancies (95% CI: 20%–39%, *I* ^2^ = 74.54%; Figure S10) and cesarean sections performed in 69% of the deliveries (95% CI: 38%–99%, *I* ^2^ = 97.57%; Figure S11). In these analyses, no publication bias was detected, except for cesarean delivery rate, where Egger's test (*p*‐value < 0.001) suggested potential bias, prompting the use of the trim‐and‐fill method. After imputing two missing studies on the right side of the funnel plot (R0), the recalculated incidence rate for cesarean delivery in EE‐FGR pregnancies was 77% (95% CI: 73%–82%).

**FIGURE 2:**
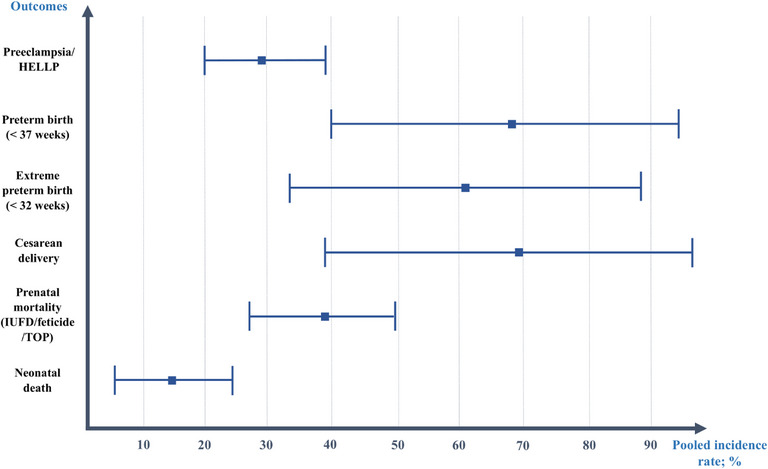
An overall view of the results of our meta‐analysis, calculating the pooled incidence rates for the primary and secondary outcomes of this study. HELLP syndrome: hemolysis, elevated liver enzyme, and low platelet syndrome; Pregnancy loss was defined by any death during antenatal period, including intrauterine fetal demise (IUFD) (<20 GA); TOP, termination of pregnancy; still birth (>20 GA); neonatal death was defined as death occurred within the first 30 days of life.

A summary of the results from our meta‐analysis of the primary and secondary outcomes is presented in Figure 2.

## 4 DISCUSSION

### 4.1 Main findings

Our meta‐analysis provides comprehensive insights into the significantly high burden of EE‐FGR. Patients with EE‐FGR face higher risks of prenatal and neonatal mortality. Our analysis revealed a pregnancy loss rate of 39%. Among live births, neonatal death within the first 30 days of life occurred at a rate of 14%. PTBs were documented in 68% of cases, with very preterm deliveries (<32 weeks) accounting for 61%, and extreme preterm deliveries (<28 weeks) occurring in 25% of cases. Additionally, maternal complications were prevalent, with 29% affected by preeclampsia or HELLP syndrome.

### 4.2 Comparison to existing literature

It is well documented that EE‐FGR substantially increases the risks of prenatal and neonatal mortality. Among live births, three out of four are delivered prematurely, often resulting in severe adverse neonatal outcomes [27]. Compared to appropriately grown for gestational age infants, those with FGR experience higher rates of complications such as bronchopulmonary dysplasia (43% vs. 26%), surgical necrotizing enterocolitis (6% vs. 0%), and retinopathy of prematurity requiring treatment (11% vs. 0%), [21]. Additionally, FGR infants face significant neurodevelopmental challenges. A systematic review found that 12% of FGR patients experienced cognitive impairment and/or cerebral palsy [28], while another study showed that 10% of them also suffering from cerebral palsy, hearing loss, and visual impairment [10]. Mortality is also higher (9% vs. 2%), with increased use of invasive ventilation (65% vs. 50%) and longer neonatal stays [21]. Our pooled results aligned with previous studies on similar populations. For instance, Lawin‐O'Brien reported significant rates of intrauterine and neonatal deaths, with 13% of live births delivered before 28 weeks [5]. Leon‐Martinez found an increased rate of neonatal composite outcomes in cases below the third percentile [29]. Carr et al. identified a perinatal mortality rate of nearly 50% in this group [30], reinforcing that EE‐FGR and subsequent PTB are associated with a broad spectrum of adverse pregnancy outcomes, including fetal and neonatal death [5, 6, 27].

Building on these findings, it is crucial to recognize the importance of parental priorities and perspectives in managing early‐onset FGR. In a study by Spencer et al., parents identified live birth, delivery before 28 weeks, and the risk of perinatal death as primary concerns and important [18]. These outcomes, which occur at alarmingly high rates in this population (50%), highlight the need for clinicians to incorporate these prognostic endpoints into their counseling and decision‐making processes.

Although attempts have been made to prevent or treat FGR by the use of interventions such as aspirin, heparin, or maternal Sildenafil, none have effectively improved growth or outcomes [5, 27]. The primary clinical intervention remains clinically indicated PTB, supported by the timely administration of maternal corticosteroids and magnesium sulfate (employed for fetal neuroprotection) to optimize neonatal outcomes. Research suggests that for each additional day spent in utero, survival rates in EE‐FGR may increase by 2% [6]. Ongoing research focused on diagnosing the underlying molecular pathogenesis and developing personalized treatment strategies, including gene and stem cell therapies, will be essential for improving outcomes in this currently untreatable condition [5, 21, 27]

### 4.3 Strengths and limitations

This meta‐analysis is strengthened by the inclusion of a large cohort of individuals with EE‐FGR and its adherence to PRISMA guidelines, which enhances the robustness and transparency of the findings. The results provide valuable conclusions and recommendations that have significant implications for clinical care and future research directions.

However, the analysis was limited by the relatively small number of eligible studies, with varying sample sizes contributing to substantial heterogeneity in reported outcomes, potentially affecting the generalizability of our findings. This heterogeneity suggests that a one‐size‐fits‐all approach may not be appropriate for counseling families or developing management strategies. Instead, clinical decision‐making should be individualized, taking into account the specific characteristics of both the mother and fetus, and future studies are needed to better define risk stratification and guide tailored interventions.

Another key limitation is the absence of a control group in most of the included studies, which restricts the ability to make direct comparisons. Additionally, all the studies were observational, which imposes its own limitations. Publication bias cannot be ruled out, as our analysis included only published studies and did not account for unpublished data. Furthermore, we excluded conference abstracts and ongoing research due to their lack of detailed methodology, rigorous peer review, and finalized results. While this approach ensured data robustness and reproducibility, it may have led to the omission of relevant findings, which should be considered when interpreting our results.

We did not have access to detailed information on the dating methods used across the datasets (e.g., last menstrual period vs. ultrasound) and were unable to distinguish between maternal and fetal indications for delivery, which may have influenced the observed outcomes. For instance, maternal complications such as preeclampsia and HELLP syndrome could have affected the timing and rationale for delivery, potentially confounding the role of FGR on pregnancy outcomes. Future studies with more detailed data on delivery indications would help clarify this distinction.

It is also important to note that the prognosis of a structurally normal, growth‐restricted fetus depends on multiple factors, including gestational age at delivery, birth weight, and cardiovascular status [4, 30]. Although this study did not assess outcomes based on delivery timing and prematurity, many observed outcomes may be attributable to prematurity rather than FGR itself.

## 5 CONCLUSIONS AND IMPLICATIONS

This study quantified the risks of adverse neonatal and maternal outcomes associated with EE‐FGR below the third percentile and between 20 + 0 and 26 + 6 weeks of gestation. Our analysis revealed that nearly half of the pregnancies resulted in either pregnancy loss or neonatal death, underscoring the severe impact of this condition. Among those who survived, more than two thirds were born preterm, indicating that even survival is accompanied by considerable health challenges. These findings are crucial for healthcare providers in counseling families, as they clarify the risks and possible outcomes, enabling more informed decision‐making and preparation. By sharing this information, we aim to help families better understand the prognosis and the critical role of vigilant monitoring and timely intervention in cases of severe FGR.

## AUTHOR CONTRIBUTIONS

Shohra Qaderi, Giulia Bonanni, Mohammadamin Parsaei, and Ehsan Rojhani were responsible for the study design, collecting data, and drafted the paper. Mohammadamin Parsaei and Giulia Bonanni were responsible for performing the analysis, and for performing and supervising the analysis. Satjeet Deol Chauhan was responsible for interpretation of data. Eyal Krispin, Dario O. Fauza, Anna L. David, Kjersti Aagaard, and Alireza A. Shamshirsaz supervised in designing the study and drafting the paper. All authors contributed to conception and design, drafting the article, and/or revising it critically and approved the final version to be published. All authors agreed to be accountable for all aspects of the work.

## CONFLICT OF INTEREST STATEMENT

The authors declare no conflicts of interest.

## ETHICS STATEMENT

This systematic review and *meta*‐analysis only used data from previous published studies.

## Supporting information



Supporting Information

## Data Availability

Data sharing is not applicable—no new data were generated.

## References

[1] Flenady, V. , L. Koopmans , P. Middleton , J. F. Frøen , G. C. Smith , K. Gibbons , M. Coory , et al. 2011. “Major Risk Factors for Stillbirth in High‐Income Countries: A Systematic Review and Meta‐Analysis.” The Lancet 377(9774): 1331–40. 10.1016/S0140-6736(10)62233-7 21496916

[2] Gordijn, S. , I. Beune , B. Thilaganathan , A. Papageorghiou , A. Baschat , P. Baker , R. M. Silver , K. Wynia , and W. Ganzevoort . 2016. “Consensus Definition of Fetal Growth Restriction: A Delphi Procedure.” Ultrasound in Obstetrics & Gynecology 48(3): 333–9. 26909664 10.1002/uog.15884

[3] Lackman, F. , V. Capewell , R. Gagnon , and B. Richardson . 2001. “Fetal Umbilical Cord Oxygen Values and Birth to Placental Weight Ratio in Relation to Size at Birth.” American Journal of Obstetrics and Gynecology 185(3): 674–82. 11568797 10.1067/mob.2001.116686

[4] Doi, K. , H. Sameshima , Y. Kodama , S. Furukawa , M. Kaneko , T. Ikenoue , and Miyazaki Perinatal Data Groups . 2012. “Perinatal Death and Neurological Damage as a Sequential Chain of Poor Outcome.” The Journal of Maternal‐Fetal & Neonatal Medicine 25(6): 706–9. 21728702 10.3109/14767058.2011.587061

[5] Lawin‐O'Brien, A. R. , A. Dall'Asta , C. Knight , S. Sankaran , C. Scala , A. Khalil , A. Bhide , et al. 2016. “Short‐Term Outcome of Periviable Small‐for‐Gestational‐Age Babies: Is Our Counseling Up to Date?” Ultrasound in Obstetrics & Gynecology 48(5): 636–41. 27854384 10.1002/uog.15973

[6] Baschat, A. A. , E. Cosmi , C. M. Bilardo , H. Wolf , C. Berg , S. Rigano , U. Germer . et al. 2007. “Predictors of Neonatal Outcome in Early‐Onset Placental Dysfunction.” Obstetrics & Gynecology 109(2 Part 1): 253–61. 17267821 10.1097/01.AOG.0000253215.79121.75

[7] Lundgren, E. M. , S. Cnattingius , B. Jonsson , and T. Tuvemo . 2001. “Intellectual and Psychological Performance in Males Born Small for Gestational Age with and without Catch‐Up Growth.” Pediatric Research 50(1): 91–6. 11420424 10.1203/00006450-200107000-00017

[8] Bardin, C. , G. Piuze , and A. Papageorgiou . 2004. “Outcome at 5 Years of Age of SGA and AGA Infants Born Less Than 28 Weeks of Gestation.” Seminars in Perinatology 28(4): 288–294. 15565789 10.1053/j.semperi.2004.08.006

[9] Sung, I.‐K. , B. Vohr , and W. Oh . 1993. “Growth and Neurodevelopmental Outcome of Very Low Birth Weight Infants with Intrauterine Growth Retardation: Comparison with Control Subjects Matched by Birth Weight and Gestational Age.” The Journal of Pediatrics 123(4): 618–24. 7692029 10.1016/s0022-3476(05)80965-5

[10] Lees, C. C. , N. Marlow , A. van Wassenaer‐Leemhuis , B. Arabin , C. M. Bilardo , C. Brezinka , S. Calvert , et al. 2015. “2 Year Neurodevelopmental and Intermediate Perinatal Outcomes in Infants with Very Preterm Fetal Growth Restriction (TRUFFLE): A Randomised Trial.” The Lancet 385(9983): 2162–72. 10.1016/S0140-6736(14)62049-3 25747582

[11] Lienhardt, A. , J.‐C. Carel , P.‐M. Preux , R. Coutant , and J.‐L. Chaussain . 2002. “Amplitude of Pubertal Growth in Short Stature Children with Intrauterine Growth Retardation.” Hormone Research 57(Suppl 2): 88–94. 12065935 10.1159/000058108

[12] Stein, C. , C. Fall , K. Kumaran , C. Osmond , D. Barker , and V. Cox . 1996. “Fetal Growth and Coronary Heart Disease in South India.” The Lancet 348(9037): 1269–73. 10.1016/s0140-6736(96)04547-3 8909379

[13] Barker, D. J. 2004. “The Developmental Origins of Chronic Adult Disease.” Acta Paediatrica 93: 26–33. 10.1111/j.1651-2227.2004.tb00236.x 15702667

[14] Spencer, R. , G. Ambler , J. Brodszki , A. Diemert , F. Figueras , E. Gratacós , S R. Hansson , et al. 2017. “EVERREST Prospective Study: A 6‐Year Prospective Study to Define the Clinical and Biological Characteristics of Pregnancies Affected by Severe Early Onset Fetal Growth Restriction.” BMC Pregnancy and Childbirth 17: 1–8. 28114884 10.1186/s12884-017-1226-7 PMC5259830

[15] Page, M. J. , J. E. McKenzie , P. M. Bossuyt , I. Boutron , T. C. Hoffmann , C. D. Mulrow , L. Shamseer . et al. 2021. “The PRISMA 2020 Statement: An Updated Guideline for Reporting Systematic Reviews.” BMJ 372: n71. 33782057 10.1136/bmj.n71 PMC8005924

[16] Proske, A. , C. Wenzel , and M. B. Queitsch . 2023. “Reference Management Systems.” In Digital Writing Technologies in Higher Education: Theory, Research, and Practice, edited by O. Kruse , C. Rapp , C. M. Anson , K. Benetos , E. Cotos , A. Devitt , and A. Shibani , p. 215–30. Cham: Springer.

[17] Ouzzani, M. , H. Hammady , Z. Fedorowicz , and A. Elmagarmid . 2016. “Rayyan—A Web and Mobile App for Systematic Reviews.” Systematic Reviews 5: 1–10. 27919275 10.1186/s13643-016-0384-4 PMC5139140

[18] Spencer, R. , K. Maksym , K. Hecher , K. Maršál , F. Figueras , G. Ambler , H. Whitwell , et al. 2023. “Maternal PlGF and Umbilical Dopplers Predict Pregnancy Outcomes at Diagnosis of Early‐Onset Fetal Growth Restriction.” Journal of Clinical Investigation 133(18): e169199. 37712421 10.1172/JCI169199 PMC10503803

[19] National Institute of Health . 2014. “Quality Assessment Tool for Observational Cohort and Cross‐Sectional Studies.” https://www.nhlbi.nih.gov/health‐topics/study‐quality‐assessment‐tools.

[20] Harrer, M. , P. Cuijpers , T. Furukawa , and D. Ebert . 2021. Doing Meta‐Analysis with R: A Hands‐On Guide. New York: Chapman and Hall/CRC.

[21] Lingam, I. , J. Okell , K. Maksym , R. Spencer , D. Peebles , G. Buquis , G. Ambler , et al. 2023. “Neonatal Outcomes Following Early Fetal Growth Restriction: A Subgroup Analysis of the EVERREST Study.” Archives of Disease in Childhood: Fetal and Neonatal Edition 108(6): 599–606. 37185272 10.1136/archdischild-2022-325285

[22] Begg, C. B. , and M. Mazumdar . 1994. “Operating Characteristics of a Rank Correlation Test for Publication Bias.” Biometrics 50(4): 1088–101. 7786990

[23] Egger, M. , G. D. Smith , M. Schneider , and C. Minder . 1997. “Bias in Meta‐Analysis Detected by a Simple, Graphical Test.” BMJ 315(7109): 629–34. 9310563 10.1136/bmj.315.7109.629 PMC2127453

[24] Duval, S. , and R. Tweedie . 2000. “Trim and Fill: A Simple Funnel‐Plot–Based Method of Testing and Adjusting for Publication Bias in Meta‐Analysis.” Biometrics 56(2): 455–63. 10877304 10.1111/j.0006-341x.2000.00455.x

[25] Mari, G. , F. Hanif , M. Kruger , E. Cosmi , J. Santolaya‐Forgas , and M. C. Treadwell . 2007. “Middle Cerebral Artery Peak Systolic Velocity: A New Doppler Parameter in the Assessment of Growth‐Restricted Fetuses.” Ultrasound in Obstetrics & Gynecology 29(3): 310–6. 17318946 10.1002/uog.3953

[26] Dap, M. , D. Allouche , E. Gauchotte , C. Bertholdt , and O. Morel . 2023. “Perinatal Outcomes of Severe, Isolated Intrauterine Growth Restriction Before 25 Weeks' Gestation: A Retrospective Cohort Study.” Journal of Gynecology Obstetrics and Human Reproduction 52(1): 102514. 36436808 10.1016/j.jogoh.2022.102514

[27] Dall'Asta, A. , and C. Lees . 2018. “Early Second‐Trimester Fetal Growth Restriction and Adverse Perinatal Outcomes.” Obstetrics & Gynecology 131(4): 739–40. 10.1097/AOG.0000000000002548 29578967

[28] Pels, A. , I M. Beune , A G. van Wassenaer‐Leemhuis , J. Limpens , and W. Ganzevoort . 2020. “Early‐Onset Fetal Growth Restriction: A Systematic Review on Mortality and Morbidity.” Acta Obstetricia et Gynecologica Scandinavica 99(2): 153–66. 31376293 10.1111/aogs.13702 PMC7004054

[29] Leon‐Martinez, D. , L. S. Lundsberg , J. Culhane , J. Zhang , M. Son , and U. M. Reddy . 2023. “Fetal Growth Restriction and Small for Gestational Age as Predictors of Neonatal Morbidity: Which Growth Nomogram to Use?” American Journal of Obstetrics and Gynecology 229(6): 678.e1–.e16. 10.1016/j.ajog.2023.06.035 37348779

[30] Carr, D. , R. Spencer , L. Waterworth , E. Dimas , and A. David . 2018. “522: Short‐Term Outcomes of Severe Early‐Onset Uteroplacental Fetal Growth Restriction Managed in a Tertiary Centre.” American Journal of Obstetrics & Gynecology 218(1): S312–S3.

