# Supplementary material for: Quantifying prenatal and postnatal mortality in extreme early onset fetal growth restriction: A systematic review and incidence analysis
Source: Pregnancy (Hoboken). 2025 May 24;1(3):e70034. doi: 10.1002/pmf2.70034 (PMC13344764; doi:10.1002/pmf2.70034)
Supplement: Supplementary file 1 [file PMF2-1-e70034-s001.docx]

**Supplementary Table 1.** Quality assessment based on NIH Quality Assessment Tool for Observational Cohort and Cross-Sectional Studies. *

| **Question/Study** | **Mari et al. ^2007^** | **Lawin-O’Brien et al. ^2016^** | **Dap et al. ^2022^** | **Rebecca Spencer, ^2023^** | **Ingran Lingam, ^2023^** |
| --- | --- | --- | --- | --- | --- |
| Was the research question or objective in this paper clearly stated? | Yes | Yes | Yes | Yes | Yes |
| Was the study population clearly specified and defined? | Yes | Yes | Yes | Yes | Yes |
| Was the participation rate of eligible persons at least 50%? | Yes | Yes | Yes | Yes | Yes |
| Were all the subjects selected or recruited from the same or similar populations (including the same time period)? | Yes | Yes | Yes | Yes | Yes |
| Was a sample size justification, power description, or variance and effect estimates provided? | No | No | No | No | No |
| For the analyses in this paper, were the exposure(s) of interest measured prior to the outcome(s) being measured? | Yes | Yes | Yes | Yes | Yes |
| Was the timeframe sufficient so that one could reasonably expect to see an association between exposure and outcome if it existed? | Yes | Yes | Yes | Yes | Yes |
| For exposures that can vary in amount or level, did the study examine different levels of the exposure as related to the outcome (e.g., categories of exposure, or exposure measured as continuous variable)? | No | Yes | No | Yes | Yes |
| Were the exposure measures (independent variables) clearly defined, valid, reliable, and implemented consistently across all study participants? | Yes | Yes | Yes | Yes | Yes |
| Was the exposure(s) assessed more than once over time? | Yes | Yes | No | Yes | Yes |
| EWere the outcome measures (dependent variables) clearly defined, valid, reliable, and implemented consistently across all study participants? | Yes | Yes | Yes | Yes | Yes |
| Were the outcome assessors blinded to the exposure status of participants? | No | No | No | No | No |
| Was loss to follow-up after baseline 20% or less? | No | Yes | No | Yes | Yes |
| Were key potential confounding variables measured and adjusted statistically for their impact on the relationship between exposure(s) and outcome(s)? | N/A | N/A | N/A | Yes | Yes |
| **Overall appraisal** | **Fair** | **Good** | **Good** | **Good** | **Good** |

**Abbreviations:** N/A = not available.

*A study will be rates as “Good” if it receives a “Yes” response for at least 80% of the applicable NIH Critical Appraisal questions, “Fair” for 50%-80%, and “Poor” for less than 50%.

**Supplementary Figure 1.** Pooled proportion of pregnancy loss outcome. (a) Forest plot, (b) Forest plot for the leave-one-out sensitivity analysis, and (c) Funnel plots for the publication bias assessment.

**(a)**

**(b)**

**(c)**

1. Begg’s test: P-value = 1.000 (no potential publication bias)
2. Egger’s test: P-value = 0.454 (no potential publication bias)

**Supplementary Table 2.** Results of the univariate meta-regression analysis for the meta-analysis of pregnancy loss rates.

| **Variable** | **Groups** | **Study number** | **β** | **95% CI** | **SE** | **z** | **P-value** | **R^2^** | **Remained I^2^** |
| --- | --- | --- | --- | --- | --- | --- | --- | --- | --- |
| Publication year | 1. Before 2020 | (n = 4) | -0.151 | -0.241, -0.062 | 0.046 | -3.31 | 0.001 | 98.16% | 3.90% |
|  | 2. After 2020 |  |  |  |  |  |  |  |  |
| Study design | 1. Prospective | (n = 4) | 0.129 | -0.391, 0.298 | 0.862 | 1.51 | 0.132 | 40.92% | 59.54% |
|  | 2. Retrospective |  |  |  |  |  |  |  |  |
| Study setting | 1. Single-center | (n = 4) | 0.059 | -0.174, 0.292 | 0.119 | 0.49 | 0.622 | 0.00% | 81.51% |
|  | 2. Multicenter |  |  |  |  |  |  |  |  |
| EE-FGR criteria | 1. EFW | (n = 4) | 0.166 | 0.079, 0.251 | 0.044 | 3.78 | < 0.001 | 100.00% | 0.01% |
|  | 2. AC |  |  |  |  |  |  |  |  |

**Abbreviations:** AC = abdominal circumference, CI = confidence interval, EE-FGR = extremely early-onset fetal growth retardation, EFW = estimated fetal weight, SE = standard error.

**Supplementary Figure 2.** Forest plots for the subgroup analysis for the pooled proportion of pregnancy loss outcome based on the year of publication.

**Supplementary Figure 3.** Forest plots for the subgroup analysis for the pooled proportion of pregnancy loss outcome based on the study design.

**Supplementary Figure 4.** Forest plots for the subgroup analysis for the pooled proportion of pregnancy loss outcome based on the EE-FGR diagnostic criteria.

**Supplementary Figure 5.** Pooled proportion of neonatal death outcome. (a) Forest plot, (b) Forest plot for the leave-one-out sensitivity analysis, and (c) Funnel plots for the publication bias assessment.

**(a)**

**(b)**

**(c)**

1. Begg’s test: P-value = 0.734 (no potential publication bias)
2. Egger’s test: P-value = 0.025 (potential publication bias)

**Supplementary Table 3.** Results of the univariate meta-regression analysis for the meta-analysis of neonatal death rates.

| **Variable** | **Groups** | **Study number** | **β** | **95% CI** | **SE** | **z** | **P-value** | **R^2^** | **Remained I^2^** |
| --- | --- | --- | --- | --- | --- | --- | --- | --- | --- |
| Publication year | 1. Before 2020 | (n = 4) | -0.145 | -0.327, 0.036 | 0.093 | -1.57 | 0.117 | 27.74% | 76.94% |
|  | 2. After 2020 |  |  |  |  |  |  |  |  |
| Study design | 1. Prospective | (n = 4) | -0.114 | -0.396, 0.167 | 0.143 | -0.80 | 0.426 | 0.00% | 90.84% |
|  | 2. Retrospective |  |  |  |  |  |  |  |  |
| Study setting | 1. Single-center | (n = 4) | -0.002 | -0.311, 0.306 | 0.157 | -0.01 | 0.988 | 0.00% | 89.97% |
|  | 2. Multicenter |  |  |  |  |  |  |  |  |
| EE-FGR criteria | 1. EFW | (n = 4) | 0.037 | -0.283, 0.357 | 0.164 | 0.22 | 0.822 | 0.00% | 91.61% |
|  | 2. AC |  |  |  |  |  |  |  |  |
| GA at delivery |  | (n = 4) | -0.042 | -0.086, 0.002 | 0.022 | -1.87 | 0.062 | 46.32% | 71.43% |

**Abbreviations:** AC = abdominal circumference, CI = confidence interval, EE-FGR = extremely early-onset fetal growth retardation, EFW = estimated fetal weight, GA = gestational age, SE = standard error.

**Supplementary Figure 6.** Forest plots for the subgroup analysis for the pooled proportion of neonatal death outcome based on the mean gestational age at birth (below and over 30 weeks).

**Supplementary Figure 7.** Pooled proportion of preterm birth (gestational age at birth < 37 weeks) outcome. (a) Forest plot, (b) Forest plot for the leave-one-out sensitivity analysis, and (c) Funnel plots for the publication bias assessment.

**(a)**

**(b)**

**(c)**

1. Begg’s test: P-value = 0.734 (no potential publication bias)
2. Egger’s test: P-value = 0.879 (no potential publication bias)

**Supplementary Figure 8.** Pooled proportion of very preterm birth (gestational age at birth < 32 weeks) outcome. (a) Forest plot, (b) Forest plot for the leave-one-out sensitivity analysis, and (c) Funnel plots for the publication bias assessment.

**(a)**

**(b)**

**(c)**

1. Begg’s test: P-value = 0.734 (no potential publication bias)
2. Egger’s test: P-value = 0.444 (potential publication bias)

**Supplementary Figure 9.** Gestational age at delivery among surviving fetuses.

This figure presents the distribution of gestational ages at delivery for fetuses that survived until birth. Gestational age categories are based on definitions provided by the original studies included in the analysis. All values represent the number of patients in each group, with "Total*" indicating the overall number of survivors in the study.

**Supplementary Figure 10.** Pooled proportion of preeclampsia/HELLP syndrome outcome. (a) Forest plot, (b) Forest plot for the leave-one-out sensitivity analysis, and (c) Funnel plots for the publication bias assessment.

**(a)**

**(b)**

**(c)**

1. Begg’s test: P-value = 1.000 (no potential publication bias)
2. Egger’s test: P-value = 0.920 (no potential publication bias)

**Supplementary Figure 11.** Pooled proportion of cesarean delivery outcome. (a) Forest plot and (b) Funnel plots for the publication bias assessment.

**(a)**

**(b)**

1. Begg’s test: P-value = 0. 296 (no potential publication bias)
2. Egger’s test: P-value < 0.001 (potential publication bias)
